# Presumed consent: licenses and limits inferred from the case of geriatric hip fractures

**DOI:** 10.1186/s12910-017-0180-2

**Published:** 2017-02-24

**Authors:** Joseph Bernstein, Drake LeBrun, Duncan MacCourt, Jaimo Ahn

**Affiliations:** 1 University of Pennsylvania, Philadelphia, USA; 2 Harvard Medical School, Boston, USA

**Keywords:** Presumed consent, Geriatrics, Hip fractures, Informed consent

## Abstract

**Background:**

Hip fractures are common and serious injuries in the geriatric population. Obtaining informed consent for surgery in geriatric patients can be difficult due to the high prevalence of comorbid cognitive impairment. Given that virtually all patients with hip fractures eventually undergo surgery, and given that delays in surgery are associated with increased mortality, we argue that there are select instances in which it may be ethically permissible, and indeed clinically preferable, to initiate surgical treatment in cognitively impaired patients under the doctrine of presumed consent. In this paper, we examine the boundaries of the license granted by presumed consent and use the example of geriatric hip fracture to build an ethical framework for understanding the doctrine of presumed consent.

**Discussion:**

The license to act under presumed consent requires three factors: patient incapacity, clinical urgency and clarity on the correct course of action. All three can apply to geriatric hip fracture. The typical patient frequently lacks capacity. Delays in initiating surgical treatment are associated with markedly increased mortality rates. Last, there appears to be consensus that surgery is the preferred treatment. Nonetheless, because there is a window of safe delay during which treating physicians can stabilize the patient, address reversible causes of cognitive impairment and identify surrogate decision makers, presumed consent should be invoked only as a method of last resort.

**Conclusions:**

A medical situation need not be characterized by risk of imminent and certain death for presumed consent to be relevant. Rather, there are two distinct windows that must be considered: the time interval in which action may be delayed without danger, and the time interval needed to obtain a better form of consent. Presumed consent is appropriate only when the latter exceeds the former.

## Background

Hip fracture is primarily a condition affecting the elderly. According to a recent review [1] of New York state data over a 12 year period, the mean age of nearly 200,000 patients who had hip fracture surgery was 79.1. Thus, it should come as no surprise that many patients presenting with this injury might lack the capacity to provide informed consent for treatment. In one examination [2] of 674 community resident patients aged 65 and older with a hip fracture, 28% had known dementia at presentation and an additional 8% had cognitive impairment first detected at the time of presentation, a finding replicated in another study [3].

The high prevalence of cognitive impairments among geriatric patients with hip fracture poses a clinically important problem, as the Western approach to geriatric hip fracture is urgent surgical treatment. In a review [4] of the 5-year experience at our institution, all 389 patients aged 60 and older admitted with a hip fracture were treated surgically. Similarly, a retrospective cohort study of 165,861 Medicare beneficiaries admitted for hip fracture in three states over a 5-year period revealed that operative management was employed for 94% of patients. In the United Kingdom, the National Hip Fracture Database 2015 Report reported a 97.8% rate of operative management in a cohort of 64,102 hip fracture patients. A separate study [5] of 1206 patients admitted with a hip fracture to one of four hospitals in New York demonstrated a 97% rate of operative management.

The urgency of initiating surgical treatment can be inferred from studies showing an association between delayed treatment and an increased risk of harm. A meta-analysis [6] of 16 studies with 257,367 patients found that patients who had did not have surgery within 48 h of admission had 41% higher 30-day mortality rates and 32% higher 1-year mortality rates. While some component of this higher rate may reflect the fact that sicker patients would be delayed, an instrumental variable analysis [7] that removed the potential selection bias of comorbidities still found that 48 h delays were associated with a 15% higher 30-day mortality rate. A separate study in Ireland [8] determined that medically fit patients who received hip fracture surgery more than 36 h after admission had longer hospital stays and higher mortality as an inpatient and at 30 days. With that in mind, hospitals in the England and Wales National Health Service have been incentivized with a so-called best practice tariff [9] to operate on patients with hip fractures within 36 h.

Given the high rate of cognitive impairment among geriatric hip fracture patients, the apparent inevitability of surgical intervention, and the benefits of expeditious treatment, a basic question arises: should geriatric patients who sustain a hip fracture and yet are unable to provide informed consent be taken to surgery under the doctrine of presumed consent? That is, if nearly all geriatric patients with a hip fracture ultimately receive surgical treatment, and if delaying surgical treatment inflicts harm, is it ethically permissible – and indeed preferable – to initiate surgical treatment in cognitively impaired but otherwise indicated patients without waiting for explicit consent to proceed?

We claim that the doctrine of presumed consent – which holds that in certain emergency situations the law allows intervention without explicit permission – can apply to the surgical treatment of geriatric hip fracture. Examining when presumed consent can be applied to geriatric hip fracture (and when it should not) defines the boundaries of the license granted by presumed consent, and thereby builds an ethical framework for understanding the doctrine.

## Discussion

### Surgical informed consent and its exemptions

For centuries, patients were asked to trust their doctors without question and to submit to treatments not only without a process of shared decision-making [10], but also without even being informed of what procedures or treatments were being proffered. Hippocrates, for example, advocated physicians to provide treatment “calmly and adroitly, concealing most things from the patient while you are attending to him” [11]. The great Spanish physician and philosopher Gregorio Maranon told patients “obey your doctor and you start getting well” [11]. Over time, the physician’s absolute authority gave way to the duty to inform, a duty first encompassing the nature of the procedure and then ultimately including the risks versus benefits, alternative treatments and consequences of treatment or no treatment. Consequently, a reciprocal procedure was established in which a treating physician discloses the appropriate information to a competent patient; the patient makes and communicates a voluntary decision regarding the acceptance or refusal of the treatment; and only with informed consent in hand, does the physician then implement the plan, acting as the patient’s agent [12, 13].

Granting consent requires the patient’s capacity to participate in the consent process. Appelbaum and Grisso [14] proposed that capacity comprises the following four features: the ability to communicate a choice; to understand relevant information; to appreciate the situation and its consequences; and to reason about treatment options. Echoing this, Van Staden and Kruger [15] argued that patients should be considered incapable of giving informed consent if a mental disorder prevents them from understanding what they consent to; if a disorder prevents them from making decisive choices; if a disorder prevents them from communicating consent; or if a disorder prevents them from accepting the need for intervention.

The presence or absence of capacity is a medical determination, but its complement, competency, is determined by the judiciary and accordingly defined by legal rubrics. In the UK, the Mental Capacity Act of 2005 [16] provides statutory guidance to clinicians. In the US, common law precedent dominates [17], although the exact contours vary between different jurisdictions of the individual states. However defined, when a lack of capacity prevents the acquisition of informed consent, other forms of permission may be needed. These substitutes include surrogate consent via health proxies, powers of attorney and next of kin, and administrative consent [18], in which consent is deemed present through an institutional process.

It may be also possible to offer treatment on the basis of *presumed consent*. As described by Veatch [19], *presumed consent* may apply when it can be claimed that a patient who cannot provide consent would indeed have provided consent if he or she had only been able to do so. A classic example of presumed consent arises in the case of cardiopulmonary resuscitation (CPR). Virtually all patients undergoing CPR have lost consciousness, and time is of the essence: the interval between observed cardiopulmonary collapse and initiation of CPR is directly associated with survival [20]. It can be assumed that most patients, absent an advance directive, who undergo spontaneous cardiac arrest would want CPR if they were given the choice.

### Standards for presumed consent

The case of CPR implies that the license to act under presumed consent requires three factors: incapacity, urgency and consensus. Foremost, the patient must be unable to give consent independently. Second, the situation must demand exigent intervention. Courts have held that if there is enough time to wait for a better form of consent without causing harm, then that substitute consent should be obtained prior to initiating any intervention [21]. Third, consent should only be presumed if, such treatment is considered to be what a “reasonable person would be expected to want” [22].

The standards for presumed consent seem to apply to the cognitively impaired patient with a hip fracture. Cognitive impairment implies the possible lack of capacity to provide consent directly. Urgency is defined by the harm imposed by waiting: delays, as noted, are associated with increased 1-year mortality by 15% or more (nontrivial gains– comparable, for example, to those offered by major cardiac procedures such as trans-aortic valve replacement for aortic stenosis [23].) And there does seem to be a consensus that surgical treatment is needed. Indeed, surgery has been chosen at rates exceeding those that CPR itself might be selected in cases of ischemic stroke [24].

Nonetheless, these three criteria – incapacity, urgency and consensus – apply only imperfectly to the case of geriatric hip fracture, and therefore the license to treat under presumed consent is accordingly limited.

## Incapacity

Cognitive impairments are prevalent among geriatric patients and it is therefore likely many patients presenting with this injury will not be able to provide consent. Indeed, the problem may be larger than might be suspected. A study by Heng [25] and colleagues found that 35% of geriatric patients with fractures tested positive for cognitive impairment after completing a cognitive assessment, and an additional 44% were unable to even complete the cognitive exam. Another study [26] of 1010 elderly hip fracture patients noted a rate of cognitive impairment of 50%.

In the case of geriatric hip fracture, some patients will present with a cognitive impediment that may be reversible with appropriate interventions. Delirium [27] secondary to sensory/environmental changes, drug effects, drug withdrawal, cardiopulmonary compromise, infection, fluid-electrolyte disturbances, pain, and endocrine abnormalities often responds to aggressive treatment [28]. Helpful steps include providing medications (e.g., anti-psychotics or analgesics) [29], withdrawing medications (e.g., benzodiazepines and anticholinergics that may cloud the patient’s mental state), repleting fluids and electrolytes, addressing sensory impairments and normalizing the environment with appropriate lighting and ambient noise [30].

The better approach to obtaining consent for geriatric hip fracture, then, might be to maintain a high index of suspicion that that the patient might not be mentally capable. A closer examination based on validated instruments to assess mental status such as the Assessment of Capacity for Everyday Decision-Making [31], Mini Mental Status Exam, Montreal Cognitive Assessment, or Mini-Cog tests may be in order [32]. Consultation with the hospital’s psychiatric consultation-liaison (CL) service, may be warranted in any question of competence. It is likewise apt for the treatment team or the CL service to periodically reassess patients for improvements in their ability to provide consent after admission, given that mental status may wax and wane in elderly patients with delirium.

## Urgency

The “before too much time has lapsed” standard is highly relevant to the issue of geriatric hip fracture. The case of CPR suggests a dichotomy: namely, cases in which instantaneous action is required, and cases in which time is allowed. This dichotomy is false, as “instantaneous” is not the proper standard. Rather, the appropriate analysis should consider two time windows: the amount of time in which inaction is likely to remain safe and the amount of time it will take to obtain consent. Presumed consent becomes permissible only when the latter exceeds the former.

In the case of CPR, for example, the window of safety might be a few minutes, whereas the acquisition of alternative consent may take hours. It is this contrast that drives the analysis, as noted in the legal case *Rogers v Sells* [21]. Here, a surgeon was found liable for not obtaining parental informed consent before amputating a 14-year-old boy’s mangled foot following a car accident. Although the court recognized the emergency situation, because the boy’s leg was neither turning black nor bleeding profusely, it was felt that there was enough “time and opportunity to talk with the parents”.

The NHS standard allows up to 36 h for definitive geriatric hip fracture treatment. That window of safety may afford sufficient time for the treating physicians to obtain consent via a more robust method than presumption. Contrasted to the urgency of CPR, 36 h may seem like an eternity. However, the time to wait is not infinite; vigilant attention to the clock must be paid lest that window close without appropriate action.

We suggest that those treating geriatric hip fracture be mindful of time. When a patient arrives at the hospital, a protocol should commence such that treatment is initiated within 36 h of injury. If the patient lacks capacity, efforts should be made to improve the patient's cognitive state and identify other decision-makers who can provide consent on behalf of the patient. If and when such efforts may fail, it would then be reasonable, we argue, to use presumed consent to ensure that the 36 h window of safety does not elapse. Although presumed consent may well be deemed a “consent of the last resort,” it should not be forgone if it is the best of all alternatives.

The contrast to the case of CPR highlights another critical issue as well: namely, the harm of inaction need not be all-or-none. With CPR, inaction will lead to certain death. In the case of geriatric hip fracture, the cost of delay is more probabilistic. Still, exposure to probabilistic risk is a cost and reducing risk confers benefit. We believe that this benefit must be included in the ethical calculus.

### Consensus

Presumed consent is predicated on the supposition that the patient would want the treatment – and that cannot be known with certainty. Perhaps a patient in need of CPR attempted suicide, and would staunchly reject any assistance if able to speak. Nonetheless, most patients in need of CPR did not attempt suicide and most would appreciate assistance – on that basis, consent can be presumed.

The exact threshold at which a consensus for intervention can be assumed is not defined. Veatch [33] has suggested a somewhat arbitrary but more definitive standard: “that professional informers should strive to provide a level of information that will satisfy 95% of those being informed.” His logic: 5% is the *p* value threshold in statistical hypothesis testing; hence a treatment that would be selected by 95% should be considered universal.

By that 95% standard, the willingness to accept surgical intervention for geriatric hip fracture should be presumed. It is worth considering, however, that the apparent consensus that surgery is needed for geriatric hip fracture may not be as robust as surmised. In a Canadian study [34], for example, more than 10% of patients were treated non-operatively. A study from Singapore [35] reported that 727 (26.4%) of 2756 hip fracture patients opted for non-surgical intervention (though it was noted that surgical management was associated with a lower complication rate and reduced length of hospital stay).

The possibility of variability in the incidence of surgical treatment further suggests that the consensus for surgery simply reflects an error in belief, albeit a widely held one. It is certainly possible that the goal of palliating [36] the patients’ suffering need not require a surgical operation every time. Application of functions such as The Nottingham Hip Fracture Score [37] –a validated predictor of 30 day mortality, among other metrics– may help identify those patients in which treatment is likely futile, simply on the basis of longevity.

Furthermore, it may not be sufficient to say that “surgery” is indicated, since the type of operation also needs to be determined as part of a process of shared decision-making [38]. Specifically, the configuration of the fracture pattern, the needs and goals of the patient, the presence or absence of degenerative joint disease, and other considerations may point to partial joint replacement, total joint replacement or fixation. Hence, it may not be enough to say “permission to operate can be presumed.” Rather, even if presumed consent must be relied upon, there should be an explicit consideration of patient-specific goals and how different operative interventions can best address those goals.

To that end, we recommend that once it is determined that the patient cannot provide consent, an ad hoc treatment committee should be convened to determine the optimal treatment of the given patient’s fracture [39]. This committee would use known information about the patient and general knowledge about patient preferences to guide its recommendation. This committee may have a broader mandate in venues like the United States, where care is less likely to be governed by protocol. Differences in venue may also suggest other context-specific priorities or norms. For example, a study from Nigeria [40] highlighted the importance of sociocultural factors in the consent process, such as the strong reliance on the extended family system. Additionally, this study acknowledged the variations not only between countries but also within countries. The authors identified regional differences in the ability to comprehend informed consent, which were driven by differences in education level and strength of religion [41].

When using population data to infer that the surgery would be desired, what matters, too, is not only the fraction of people that would accept the treatment, but the overall costs and benefits. It needs to be established not only that 95% of patients would submit to surgery, but that the costs (disutility) to the other 5% would not negate the potential gains. Regarding geriatric hip fractures, there is, to our knowledge, a paucity of data to drive a true expected utility analysis—namely, what is truly valued by patients and the risks and costs they will tolerate in search of positive outcomes. Nevertheless, studies that clarify these issues have been done in other domains [42] and it stands to reason that they can be done for geriatric hip fracture as well. Indeed, Alolabi et al. [43] developed a decision board for the surgical management of displaced femoral neck fractures and found that, using parameters derived from four randomized controlled trials, an overwhelming majority of patients preferred total hip replacement to hemiarthroplasty. Additional work in this area, examining the boundary conditions for the typical patient’s change in plan, may help guide decision making here, both by patients directly, and by surrogates who may be called to make decision on patients’ behalf.

Similarly, beyond optimizing clinical outcomes and preventing mortality, the surgeon should consider how a given intervention will affect long-term health-related quality of life. Hip fracture is associated with a significant loss of health-related quality of life, and this effect is more significant among patients with cognitive impairment [44]. Whenever possible, patient-specific priorities should be an important consideration of the treatment committee.

## Conclusion

**Fig. 1:**
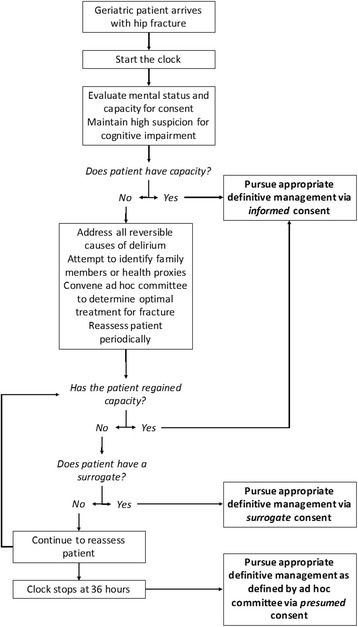
The authors’ approach to managing consent in the case of geriatric hip fracture

The case of geriatric hip fracture opens a window to examine the license and limits of presumed consent. A medical situation need not be characterized by a risk of imminent and certain death for presumed consent to be relevant. Rather, we argue that waiting for a better form of consent must be balanced with time-dependent probabilistic risk of harm. In the case of geriatric hip fracture, there is a role for presumed consent, though it is not the preferred method, to be sure (see Fig. 1 and Table 1).

**Table 1:** A rationale for the authors’ approach to consent in the case of geriatric hip fracture

Action	Rationale
1. Start the clock. Ensure that the question of capacity is fully answered to allow treatment within the 36 h window of safety.	There is a strong association between delayed treatment and an increased risk of harm
2. Assess mental status closely.	Many patients with geriatric hip fracture may have a cognitive impairment
3. If capacity is not assured:	
a. Attempt to address the causes of cognitive impairment (via steps noted above), and reassess patient periodically to monitor effects of those efforts.	Many cases of cognitive impairment may be due to a reversible delirium
b. Attempt to identify family members, caregivers, health proxies, etc. who can provide surrogate consent.	Surrogate consent is preferable to presumed consent
c. Convene an ad hoc treatment committee to determine, absent patient-provided consent, the ideal treatment of the given patient’s fracture.	A committee comprising members with the relevant expertise in the medical, surgical, social and functional issues will be able to best select the treatment
4. Conduct the necessary pre-operative medical workup so that the patient is able to undergo surgery as soon as either consent is achieved or 36-h window closes.	Although many patients can be readied for surgery within 36 h, there may be necessary medical evaluations and interventions to assure safe care. Waiting for improved capacity or a surrogate decision maker need not be wasted time
5. By the 36th hour, if the patient is ready for surgery in all other ways, and if the patient cannot provide informed consent, and if substitute consent has not yet been employed, treat as suggested by an ad hoc committee under presumed consent.	The risk of waiting longer outweighs the potential gains, especially if steps 1–4 are taken

Cases of geriatric hip fracture are very common: approximately 350,000 per year in the US and UK alone. Assuming consent is an issue in just 15% of these patients—and this number may be low, but allows for the cases of patients retaining capacity despite mild cognitive impairment—that represents 50,000 cases each year. Because of that substantial public health burden, capacity and consent in geriatric hip fracture thus deserve heightened awareness and additional study. Future work should focus on investigating capacity and methods to improve it, as well as obtaining valid information on what patients really want, seen in context of what physicians can really offer them.

## Abbreviations

CL: Consultation-liaison
CPR: Cardiopulmonary resuscitation
